# Complete genome sequencing, molecular and antigenic characterization of duck hepatitis A virus type 1 isolated in Benha, Egypt

**DOI:** 10.1186/s12917-025-05010-5

**Published:** 2025-10-03

**Authors:** Ibrahim Moharam, Mohamed A. Abaza, Norhan W. Khalil, Ehab M. El-Nahas, Ayman S. El-Habbaa, Fares Z. Najar, Medhat Radi, Eman M.S. El Nagar, Lamia Omar, Maha A.N. Gamal, Fouad S. El-Mayet

**Affiliations:** 1 Department of Bird and Rabbit Medicine, Faculty of Veterinary Medicine, University of Sadat City, Sadat City, Minoufiya Egypt; 2 Avian and Rabbit Diseases Department, Faculty of Veterinary Medicine, Benha University, Benha, Egypt; 3 Department of Virology, Faculty of Veterinary Medicine, Benha University, Benha, Egypt; 4 High-Performance Computing Center (HPCC), Oklahoma State University, Stillwater, OK USA; 5 Pest Physiology Department, Plant Protection Research Institute, ARC, Giza, Egypt; 6 Veterinary Serum and Vaccine Research Institute (VSVRI), Agricultural Research Center (ARC), Cairo, Egypt; 7 Central Laboratory for Evaluation of Veterinary Biologics (CLEVB), Agricultural Research Center (ARC), Cairo, Egypt

**Keywords:** NGS, DHAV-1, Ducklings, Phylogenetic analysis, Escape mutation, Egypt

## Abstract

**Background:**

Duck virus hepatitis (DVH) is highly fatal disease that predominantly affects young ducklings, causing substantial losses due to its high morbidity and mortality rates. A severe outbreak occurred in young Pekin ducklings on a commercial farm in Benha, Egypt.

**Results:**

The affected birds exhibited neurological signs, including lethargy, ataxia, and opisthotonus, leading to a high mortality rate. The livers and kidneys of ducklings showed various degrees of gross and histopathological lesions. Virus isolation trials in embryonated duck eggs revealed the characteristic greenish discoloration of the allantoic fluid, along with hepatitis and embryonic mortality. Furthermore, RT-PCR confirmed the presence of suspected duck hepatitis A virus type 1(DHAV-1). This study presents the first complete genome sequence of DHAV-1 from Egypt using next generation sequencing (NGS). Sequence analysis revealed that DHAV-1 exhibits the characteristic genomic organization of *Avihepatovirus*. The whole nucleotide sequence of Du/Egy/Benha/2020/DHAV-1 showed a high similarity to viruses isolated from Hungary in 2004, with a 99.9% identity in both the complete genome and structural genes (VP0, VP3, VP1). Antigenic analysis revealed a unique escape mutation, S178Y, related to conserved antigenic determinants on VP1 of DHAV-1 isolate. The cross-neutralization assay was utilized to assess the antigenic diversity between the field strain and the locally used live attenuated vaccine strain.

**Conclusion:**

The results revealed minimal antigenic variation, highlighting the potential for immune evasion. These findings suggest that the currently administered vaccines in Egypt remain effective in controlling DHAV-1 infections. However, continuous surveillance is essential to monitor any emerging genetic or antigenic changes that could compromise vaccine efficacy in the future.

**Supplementary Information:**

The online version contains supplementary material available at 10.1186/s12917-025-05010-5.

## Background

Duck virus hepatitis (DVH) is a highly contagious and fatal disease affecting young ducklings, primarily those under three weeks of age. It is associated with severe liver lesions, high morbidity, and mortality. It belongs to duck hepatitis A virus (DHAV), genus *Avihepatovirus*, *Picornaviridae* family [1] DHAV is traditionally classified into three types: I, II, and III. DHAV type I consists of three distinct genotypes and also serotypes. They are classified as DHAV-1, DHAV‐2 and DHAV‐3 which are genetically and geographically distinct. The most prevalent and virulent genotype is the DHAV-1 and formerly known as duck hepatitis virus type 1. Some studies classified the DHAV type 1 into DHAV-A, DHAV-B and DHAV-C genotypes [2]. While DVH type II is caused by duck astrovirus type-1 (DAstV-1), a member of the *Astroviridae* family. It was recorded in United Kingdom and China targeting ducklings of 10 days and six weeks of age. The affected ducklings have pathohistological changes that be similar to the DHAV-1 infection [3] DVH type III was originally classified as a picornavirus, but recently it was reclassified also as an astrovirus and was named duck astrovirus type 3 (DAstV‐3) [4].

DVH is clinically, characterized by high morbidity in ducklings showed nervous signs which represented in ataxia, and loss of balance are easily observed together with spasmodic kicking of both legs and later approach death with opisthotonus or head drawn back [5] Although live and inactivated vaccines are available, Duck Virus Hepatitis (DVH) remains a severe problem among young ducks, notably in Egypt. The first reported case of duck hepatitis A virus (DHAV) in Egypt occurred in 1969 [6] Several recent studies have investigated DHAV-1 in duckling flocks across various farms in Egypt. A study conducted between 2012 and 2014 across 46 commercial duck farms examined ducklings exhibiting clinical signs consistent with DVH infection. The findings confirmed that all duck breeds, including Pekin, Muscovy, Mallard, and Green-Winged, were susceptible to the disease. Phylogenetic analysis revealed a genetic relationship between Egyptian strains and the Asian DHAV-1 serotype viruses. Additionally, DHAV-3 was first reported in ducklings in Sharkia, Egypt, with an infection rate of 27.8% [7, 8]. In recent years, DHAV-1 and DHAV-3 serotypes have been isolated and identified across various regions of Egypt [9–13]. Notably, for the first time, co-circulation of both serotypes was detected in Pekin ducklings from six governorates, which exhibited neurological signs and high mortality rates [14].

Eighteen samples from Pekin and Mallard ducklings (3–11 days old) across six Egyptian governorates were tested for DVH. Phylogenomic analysis identified five positive samples, all belonging to the DHAV-1 VP1 gene [15]. Recent studies have revealed that the rapid death of young ducklings can be attributed to the onset of a severe cytokine storm induced by viral infection [16, 17].

Duck virus hepatitis continues to be widespread in China, causing substantial economic losses to the duck farming industry. Although the introduction of the DHAV-1 live attenuated vaccine in 2013 significantly reduced the transmission of the virus, recent years have seen the emergence of new challenges in its prevention and control [18]. Epidemiological studies across multiple provinces in mainland China reveal that DHAV-1 remains prevalent, with the genetic evolution of circulating strains closely linked to distinct geographical regions [19].

DHAV has a positive-sense single-stranded RNA genome approximately 7,800 bases long. The genome incorporates open reading frame (ORF) that codes for an inactive precursor protein. After a series of cleavage processes, the precursor protein transformed to the structural protein P1, which subsequently transformed to the capsid proteins VP0, VP1, and VP3. Furthermore, it produces non-structural proteins 2 A, 2B, and 2 C in the P2, in addition to the proteins 3 A, 3B, 3 C, and 3D in the P3 [1]. VP0 is additionally subdivided into VP2 and VP4. The VP1 protein is a receptor binding protein which plays a vital role in virus virulence and immunogenicity. Finally, the C-terminal of the VP1 protein gene of DHAV contains two hypervariable regions (HVRs), namely HVR1 amino acid residues 180–194 and HVR2 amino acid residues 205–219, which have been related to virulence differences among DHAV-1 strains [20]. Several studies have identified antigenic sites of DHAV-1 using monoclonal antibodies to map its neutralizing epitopes. A specific region on the VP1 protein (^173^LPAPTS^178^) was recognized as a key neutralizing epitope using monoclonal antibody 2D10. The hexapeptide LPAPTS exhibited the strongest binding affinity to 2D10 and interacted with DHAV-1-positive duck serum, highlighting its potential role in immune recognition [21]. Also, a unique epitope on the VP3 protein of DHAV-1 was identified using monoclonal antibody 3B7, which targets B cells. The minimal epitope, ^205^PSNI^208^, exhibited high conservation among DHAV-1 strains but differed in DHAV-2 and DHAV-3. However, the precise functional role of these epitopes remains undefined. Another epitope ^75^GEIILT^80^ showed weak neutralizing activities to both DHAV-1 and DHAV-3 while not with DHAV-2 VP1 [22].

Although DHAV-1 viruses belong to the same serotype, genetic diversity can impact vaccine efficacy. Kim et al., demonstrated that DHAV-1 vaccines from group 1 exhibited reduced neutralization capacity against a divergent DHAV-1 strain from group 3. The authors attributed this limited cross-protection to weaker neutralizing activity against heterologous variants [23]. These findings may explain the persistence of emergent DHAV-1 group 3 and 4 field strains despite widespread use of group 1 and 2 vaccines in China, as well as the continued spread of Group 4 strains among ducks vaccinated with group 1 vaccines in Egypt. Similarly, Wang et al., (2015), reported that virus neutralization assays assess functional antibodies induced post-vaccination, offering valuable insights into cross-reactivity and potential cross-protection between vaccine strains and virulent DHAV-1 variants.

In this study, we isolated duck hepatitis A virus type 1 (DHAV-1) from clinical samples collected from an infected duck flock in Benha, Qalyubia Governorate, in 2021. The complete genome sequence was determined using next-generation sequencing (NGS). Furthermore, genetic and antigenic characterization was conducted to evaluate the antigenic relatedness of the isolate to existing vaccinal strains.

## Results

### Clinical, postmortem and histopathological findings

**Fig. 1:**
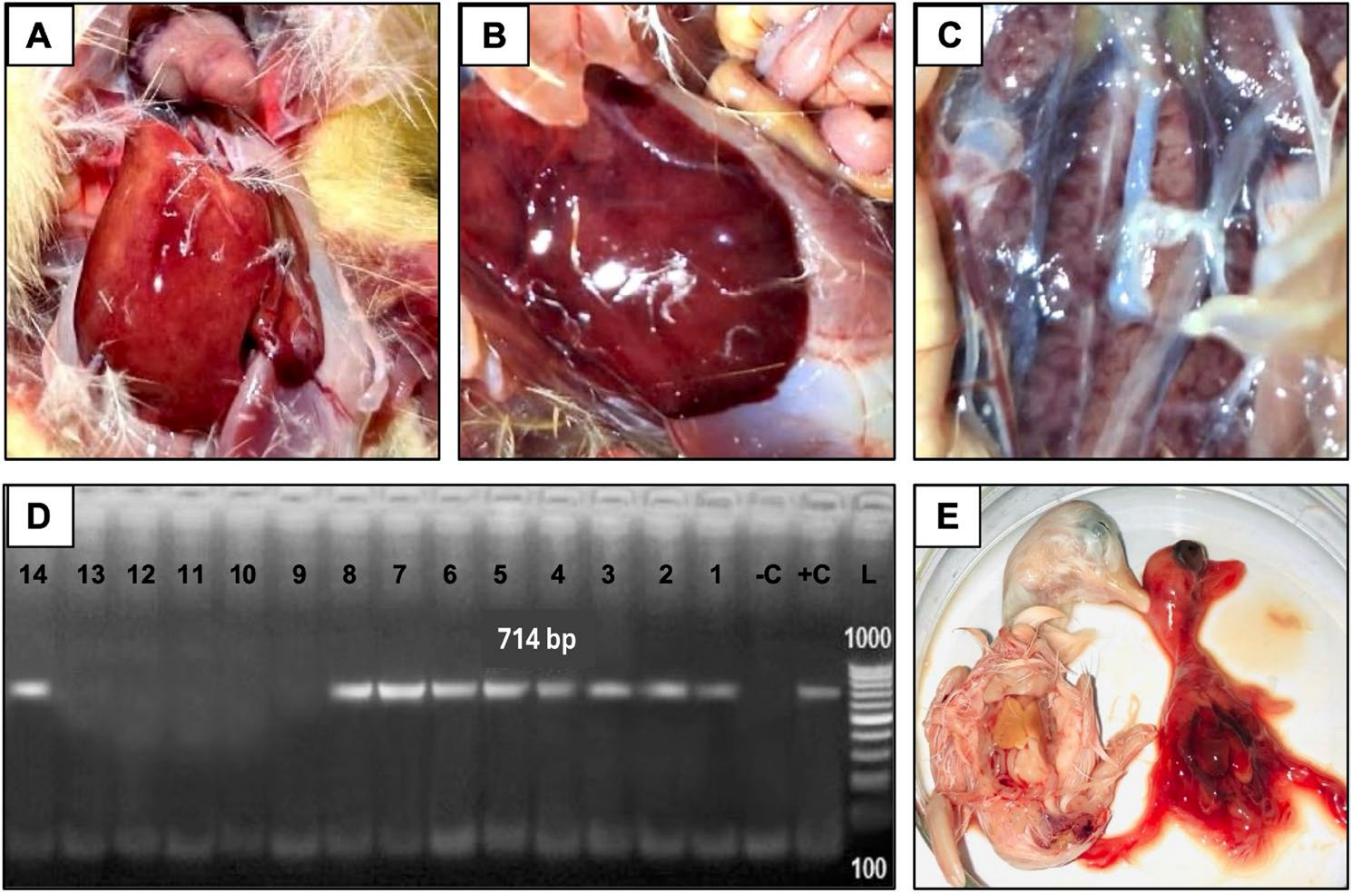
Representative images of gross lesions in clinical samples from deceased ducklings. **A** & **B **Show liver with a mottled appearance, indicating hepatitis, along with diffuse ecchymotic hemorrhages. **C **Reveals renal congestion and a swollen, hemorrhagic kidney. **D **Agarose gel electrophoresis showing RT-PCR results. L: DNA ladder, +C: positive control, -C: negative control, lanes (1–8 and 14): samples with representative positive amplification signals (amplicon size is 714 bp), Lanes(9–13): negative samples. **E **Macroscopic examination of duck embryos at 3–4 days post-inoculation with suspected duck hepatitis A virus. Inoculated embryo (right, R) exhibited signs of congestion, stunting, dwarfing, and hemorrhaging in the liver compared to normal embryo with normal liver (left, L)

A significant outbreak of a highly lethal and acute disease was reported in one-week-old Pekin ducklings at a commercial farm in Benha, Egypt. The affected flock had not been vaccinated against Duck Hepatitis A Virus Type 1 (DHAV-1). Clinical signs included depression, lethargy, ataxia, and opisthotonus, with high mortality rates. Necropsy revealed severe petechial haemorrhages on the liver (Fig. 1A & B), while the kidneys showed swelling, and congestion (Fig. 1C). Histopathological analysis of the livers demonstrated acute hepatitis, marked by multifocal hepatocyte necrosis and degeneration. The kidney examination revealed acute tubular necrosis characterized by widespread necrosis of tubular epithelial cells. The renal interstitium showed significant infiltration by mononuclear inflammatory cells.

### Viral detection in clinical samples and isolation on EDEs

DHAV-1 was identified using RT-PCR targeting the VP1 gene, with amplification yielding the expected 714 bp amplicon in tested samples, confirming DHAV type 1 infection (Fig. 1D). RT-PCR-positive samples were then used for virus isolation via inoculation into embryonated duck eggs (EDEs). Embryonic mortality occurred within 5 to 7 days post-inoculation. Notable pathological changes in the inoculated embryos included delayed development, extensive subcutaneous haemorrhages throughout the body, and significant oedema, particularly in the abdominal region. The livers of deceased embryos exhibited swelling, congestion, and necrosis. Embryos in the negative control group, inoculated with physiological saline, remained viable and showed no pathological lesions or abnormalities (Fig. 1E).

### Metagenomic next-generation sequencing (mNGS) analysis and genetic characteristics

**Fig. 2:**
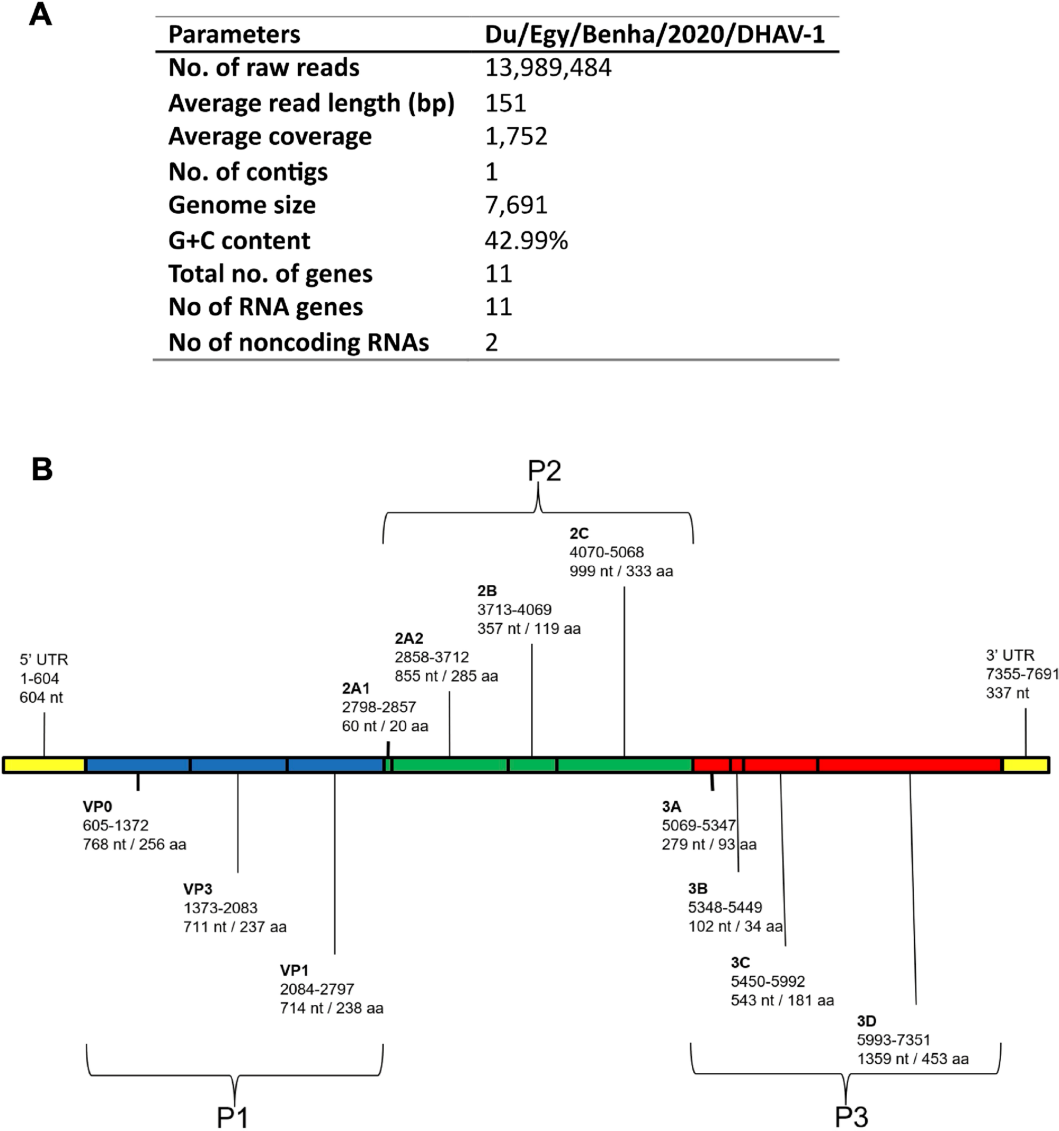
**A **Basic characteristics of the whole-genome sequencing (NGS), assembly, and annotation of strain Du/Egy/Benha/2020/DHAV-1 (Accession No: OR738706) reported in the present study. **B **Schematic depicting the organization of the duck hepatitis A virus 1 (DHAV-1)

The NGS sequencing process produced a total of 13,989,484 reads from DHAV-1, all of which were aligned in a single contig. The mean read length was 151 base pairs (bp), and the size of the genome was 7,691 bases. The genome contained 11 genes and two noncoding RNAs (UTRs). The overall G + C content of the genome is 42.99% as indicated in (Fig. 2A). The arrangement and makeup of the genome were clearly depicted in (Fig. 2B). The isolate was identified as Duck/Egypt/Benha/2020/duck hepatitis A virus type 1 (Du/Egy/Benha/2020/DHAV-1) and the final genome and its annotation were deposited in GenBank under the accession number OR738706.1.

### Phylogenetic analyses and sequence comparisons of the Du/Egy/Benha/2020/DHAV-1

Relationship distances were calculated between Du/Egy/Benha/2020/DHAV-1 and other closely related viruses retrieved from the GenBank, specifically focusing on viral genes.

**Fig. 3:**
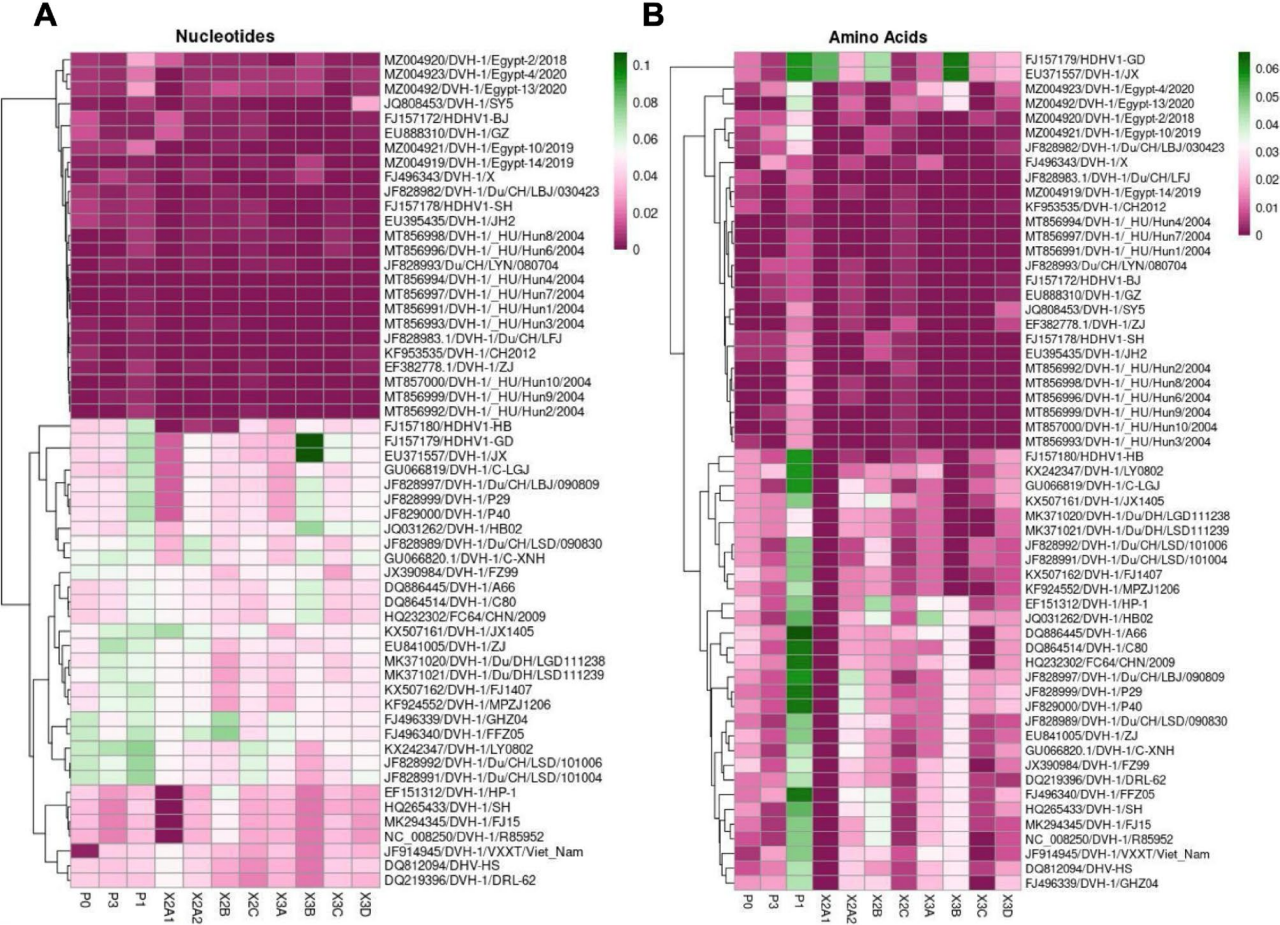
Heatmap of nucleotide (**A**) and amino acids (**B**) composition comparison of Du/Egy/Benha/2020/DHAV-1 with DHAV-1 strains. The heatmap illustrates the nucleotide or amino acids variability among strains, with clustering based on sequence similarity. Rows represent individual DHAV-3 strains (annotated with GenBank accession numbers and strain names), while columns correspond to specific nucleotide or amino acid positions across the analyzed viral genes. The color gradient from red (low) to green (high) indicates the proportion of nucleotide or amino acids occurrence at each position, highlighting patterns of conservation and variation

The Isolate Du/Egy/Benha/2020/DHAV-1 displayed 99.95% similarity with the European strains characterized from Hungary in 2004, specifically identified as MT856994/DVH-1/HU/Hun4/2004 at whole genome level. The connection at the VP0 gene level showed a similarity of 99.9% at the nucleotide level and 100% at the amino acid level with MT856994. The JF828993 strain, from China in 2008, exhibited a comparable association. The gene VP3 shows a 100% identity with the MT856994 strain, both at the nucleotide and amino acid levels. Two isolates from China, JQ808453 isolated in 2008 and KF953535 isolated in 2012, had comparable results. The gene VP1 exhibited a nucleotide similarity of 99.9% and an amino acid similarity of 99.6% with the Hungarian isolate MT856994. Regarding the non-structural genes Du/Egy/Benha/2020/DHAV-1 is closely related to viruses isolated from Hungary (MT857000, MT856998, MT856999, MT856997, MT856993, MT856991, MT856994, MT856996, MT856992). It is also related to viruses isolated from China, such as JQ808453, JF828993, EF382778, JF828983, FJ496343, KF953535, FJ157178, FJ157172, EU395435, EU888310) or viruses isolated from Egypt as (MZ004920 and MZ004919). Detailed relationships were illustrated in (Fig. 3A&B).

**Fig. 4:**
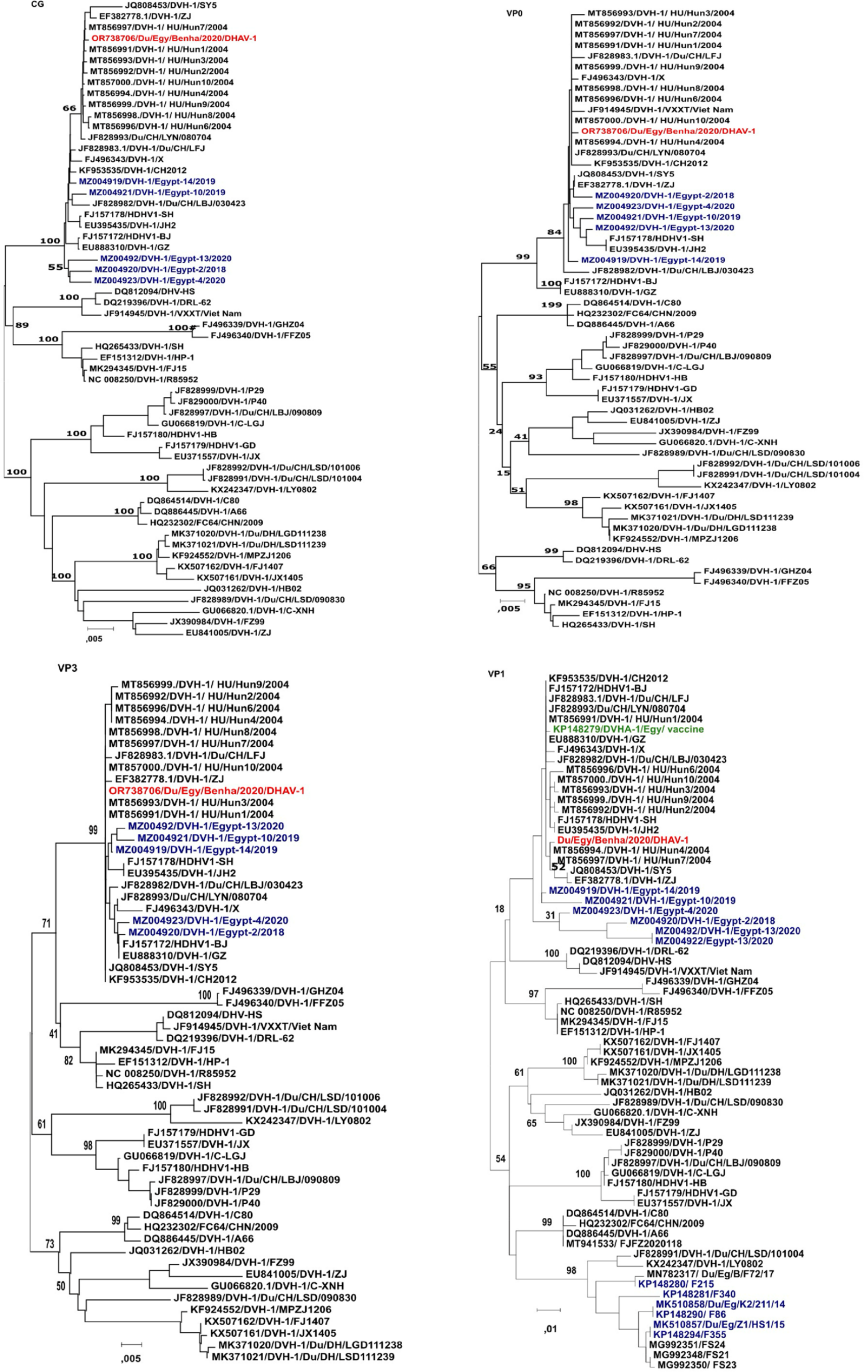
Phylogenetic analysis of Du/Egy/Benha/2020/DHAV-1 based on nucleotide sequences of the complete genome (CG) and viral structural genes. Each tree was constructed using the neighbor-joining method, with branch lengths scaled to the number of substitutions per site (indicated by the scale bar). Bootstrap values (in percentages) from 1,000 replicates are shown at the major branch points. The strain Du/Egy/Benha/2020/DHAV-1/OR738706.1 (highlighted in red) is positioned within the corresponding clades for each gene. GenBank accession numbers and strain designations are indicated for all sequences

Phylogenetic analysis at the nucleotide level showed that Du/Egy/Benha/2020/DHAV-1 clustered with Hungarian viruses from 2004 and several Chinese isolates (Fig. 4). Similarly, the VP0 and VP3 genes grouped with Hungarian and Chinese strains, along with a Vietnamese isolate in the VP0 cluster. Additionally, the VP1 gene was closely related to Hungarian, Chinese, and one Egyptian isolate (Fig. 4). These results suggest that Du/Egy/Benha/2020/DHAV-1 may represent a distinct introduction of DHAV-1 viruses into Egypt. While it was clear that there were two different introductions that were coloured with blue that had resulted in horizontal virus infections. Furthermore, although the vaccine strain (coloured with green) was clustered within the same genetic group as the field isolate based on VP1 phylogenetic analysis, but it was not directly related to it (Fig. 4).

### Analysis of the potential antigenic determinants in VP1 and VP3 protein of Du/Egy/Benha/2020/DHAV-1

**Fig. 5:**
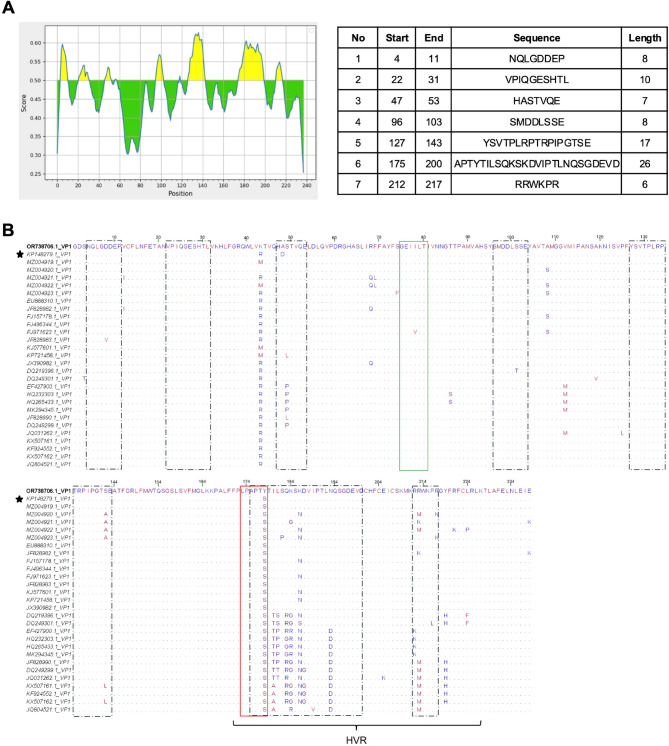
**A **Analysis of antigenic determinants on the VP1 protein of DHAV-1 is shown with residue positions on the X-axis and residue scores on the Y-axis. Residues with scores exceeding the default threshold (0.5) are identified as antigenic determinants and highlighted in yellow on the graph. Additionally, key peptides are detailed in the accompanying table on the right. **B **Alignment of the deduced amino acid sequences of VP1 surface protein of Du/Egy/Benha/2020/DHAV-1 isolate from Egypt compared with 28 DHAV-1 strains retrieved from the GenBank indicates a site at which the amino acid residue is identical to that of the Egyptian strain. The major immunodominant sites of the VP1 protein are indicated by black dashed squares, while previously identified epitopes are highlighted in green and red squares. The vaccine strain used in Egypt is marked with an asterisk

The analysis of the VP1 surface protein from the Egyptian DHAV-1 isolate (Du/Egy/Benha/2020/DHAV-1) identified several conserved residues when compared with 28 DHAV-1 strains retrieved from GenBank. These conserved regions, representing major immunodominant sites, are likely critical for structural integrity and host immune recognition. Due to the limited availability of previously characterized B-cell epitopes on VP1, antigenic determinants were analyzed using the IEDB Analysis Resource software. Regions with residue scores exceeding the 0.5 threshold were identified as antigenic, revealing seven key peptides (Fig. 5A) associated with these sites marked with dashed black squares (Fig. 5B). However, the previously recognized peptide GEIILT (6 amino acids, positions 75–80), marked with a green square, was not among them. This peptide, identified as a common epitope in both DHAV-1 and DHAV-3 [22] showed no differences compared to the Du/Egy/Benha/2020/DHAV-1 isolate (Fig. 7B), underscoring its conserved nature across DHAV genotypes. Interestingly, the peptide LPAPTS (6 amino acids, positions 173–178), previously identified by Wu et al., as a VP1-specific linear B-cell epitope recognized by the neutralizing monoclonal antibody 2D10, overlapped with one of the seven aforementioned peptides by four amino acids [21]. Among the 28 analysed strains, this peptide was entirely conserved, except in the Du/Egy/Benha/2020/DHAV-1 isolate, which exhibited a unique S178Y mutation. Notably, this mutation was exclusive to Epitope 2D10, suggesting a potential escape mutation that may affect antibody recognition and neutralization efficacy. While many peptides were highly conserved among DHAV-1 strains, significant variations were observed in certain regions, particularly between the Egyptian Du/Egy/Benha/2020/DHAV-1 isolate and other strains. Notable peptide variations included APTYTILSQKSKDVIPTLNQSGDEVD (26 amino acids, positions 175–200), RRWKPR (6 amino acids, positions 212–217), and the previously characterized LPAPTS (6 amino acids, positions 173–178), which notably falls within the hypervariable region (HVR, positions 172–218) of VP1 in DHAV-1 [23].

**Fig. 6:**
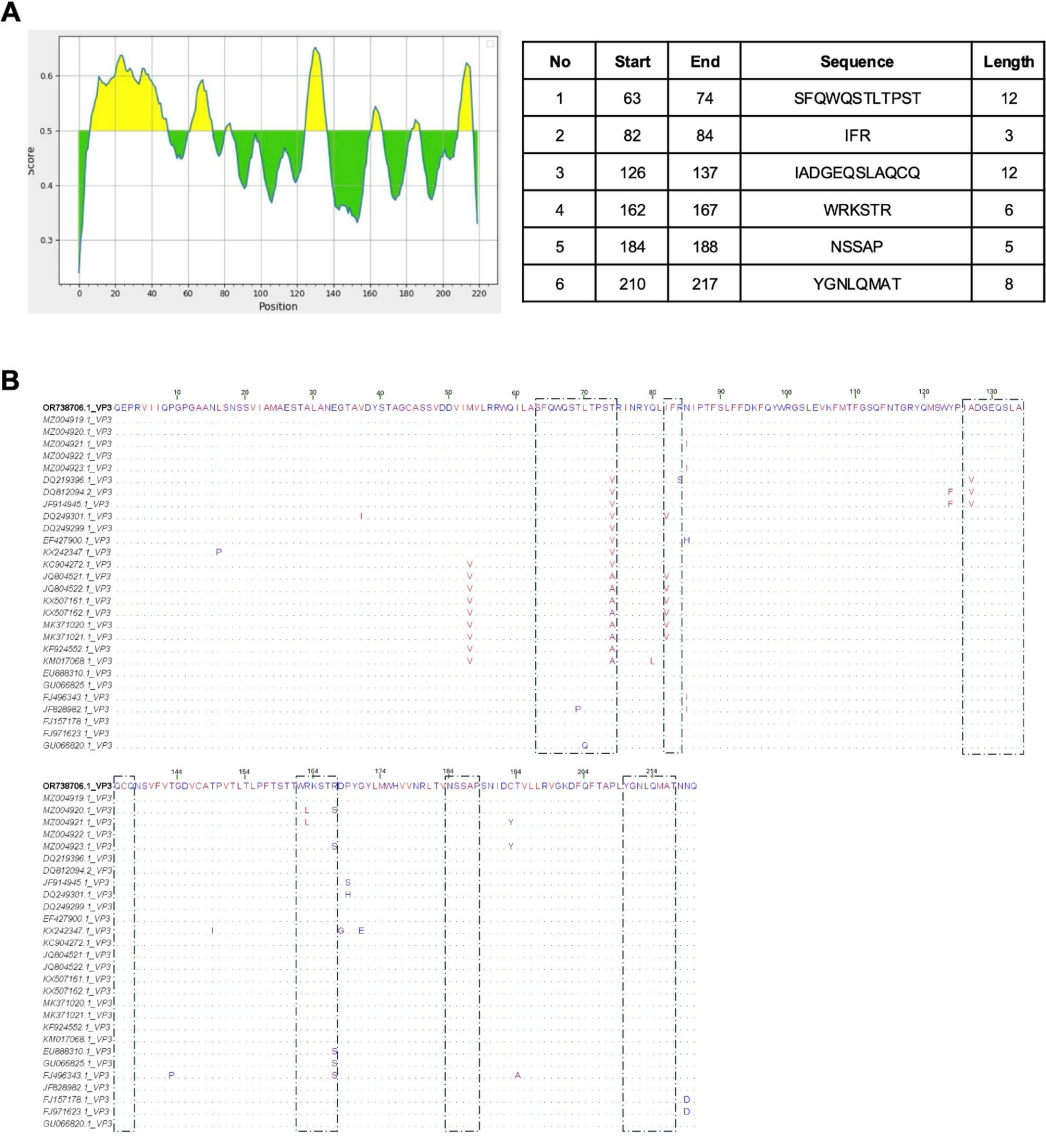
**A **Analysis of antigenic determinants on the VP3 protein of DHAV-1 is shown with residue positions on the X-axis and residue scores on the Y-axis. Residues with scores exceeding the default threshold (0.5) are identified as antigenic determinants and highlighted in yellow on the graph. Additionally, key peptides are detailed in the accompanying table on the right. **B **Alignment of the deduced amino acid sequences of VP3 surface protein of Du/Egy/Benha/2020/DHAV-1 isolate from Egypt compared with 28 DHAV-1 strains retrieved from the GenBank indicates a site at which the amino acid residue is identical to that of the Egyptian strain. The major immunodominant sites of the VP3 protein are indicated by black dashed squares

Antigenic determinants on the VP3 of DHAV-1 were also analyzed using the IEDB Analysis Resource. Regions with residue scores exceeding the 0.5 threshold were identified as antigenic, revealing six key peptides (Fig. 6A) associated with these sites marked with dashed black squares (Fig. 6B). Almost all peptides were highly conserved among DHAV-1 strains, with no significant variations observed in specific regions through amino acid alignment, particularly between the Egyptian Du/Egy/Benha/2020/DHAV-1 isolate and other strains.

### Cross neutralization between DHAV-1 virulent isolate and DHAV-1 vaccine strain

The observed mutation pattern, particularly within the HVR, may contribute to immune evasion, potentially reducing the neutralization efficacy of antibodies derived from the vaccine strain against virulent field strains. To assess this, cross-neutralization assay was performed to evaluate the impact of these variations on vaccine efficacy.

The cross-neutralization assay revealed variations in the neutralizing antibody responses against the DHAV-1 vaccine and field strains. Antibodies raised against the vaccine strain exhibited a log₂ neutralizing titer of 2^6^ (64) against the homologous vaccine strain and 2^8^ (256) against the heterologous field isolate. On the other hand, antibodies generated against the field isolate demonstrated a log₂ neutralizing titer of 2^7^ (128) against both the vaccine and field strains, suggesting a broader but relatively balanced neutralization capacity. Moreover, these findings exhibited high cross-reactivity with the homologous vaccine strain a value of *R* ≥ 0.5 signifies a minor difference between the two strains.

## Discussion

Duck hepatitis A viruses are the primary etiological agents of duck virus hepatitis, a highly infectious and serious disease that primarily impacts young ducklings. The effect is especially significant in the Egyptian duck industry, where DVH represents a substantial risk to duck farming enterprises. Efficient management and control strategies are essential to alleviate the adverse effects of this disease on the poultry sector [24]. The outbreak of DHAV-1 in one-week-old Pekin ducklings, highlights the critical requirement for effective vaccination programs in commercial duck farming. Clinical signs such as depression, lethargy, ataxia, and opisthotonus, along with necropsy findings of petechial haemorrhages on the liver and renal swelling, are aligned with previous records of DHAV-1 infections [5, 25]. Histopathology revealed acute hepatitis with hepatocyte necrosis, karyorrhexis, and cytoplasmic vacuolation, along with kidney lesions marked by tubular epithelial necrosis and mononuclear infiltration, highlighting the systemic impact of the virus. These findings align with the known pathogenesis of DHAV-1, which induces severe hepatic damage [5]. The detection of DHAV-1 via RT-PCR targeting the VP1 gene, followed by virus isolation in embryonated duck eggs, confirms its presence in the affected flock. Pathological changes in inoculated embryos indicate the virulence of DHAV-1, aligning with previous studies documenting similar embryonic pathologies associated with DHAV-1 infection [15, 26].

Recent studies reporting the isolation of DHAV-1 from duck embryos suggest the potential for vertical transmission from breeding ducks to their offspring [27]. Due to the importance of vertical transmission, eggs were sourced from a farm with strict hygiene practices. Control embryos injected with sterile PBS showed no signs of infection and appeared normal.

The genomic analysis revealed high degree of homology between Du/Egy/Benha/2020/DHAV-1 and the Hungarian strain MT856994/DVH-1/HU/Hun4/2004 with 99. 9% identity at the nucleotide level, and this homology was equally observed in the structural genes such as VP0, VP3, VP1 across different geographical regions [1]. Further phylogenetic analysis showed that Du/Egy/Benha/2020/DHAV-1 strain is most closely related to DHAV-1 strains isolated from China (JQ808453/DVH-1/SY5, KF953535/DVH-1/CH2012). These results imply the probable descent from a single ancestor of all these strains, which means that such subtypes of DHAV-1 circulate globally and remain genetically similar to each other at present. Similar findings were reported by Feher et al., who highlighted that DHAV-1 strains are highly phylogenetically clustered based on their geographic regions [28]. The phylogenetic analysis suggests the possibility of a new introduction or circulation of the existent DHAV-1 strain in Egypt. This is supported by our findings, as the Du/Egy/Benha/2020/DHAV-1 strain is closely related to the Egyptian isolate MZ004919/DVH-1/Egypt-14/2019 [15] consequently, new DHAV-1 strains were identified in Egypt and could pose a threat to the poultry industry in the region. Therefore, continuous tracking of DHAV-1 in Egypt is crucial to understand the dynamics of its spread and to implement effective measures for controlling the virus.

The antigenic determinants analysis showed that 15 of the 16 amino acids in epitopes 4F8, 2D10, and 3B7 are conserved in Du/Egy/Benha/2020/DHAV-1 and other worldwide strains. This indicate that the epitopes are well conserved, and this aligned with the study made by [22] who pointed out that DHAV-1 epitopes are stable which is advantage for development of vaccines. However, epitope 2D10 exhibits a novel strain mutation, S178Y, which may contribute to immune evasion. This mutation suggests that the virus could develop mechanisms to evade host immunity, a phenomenon observed in studies on other picornaviruses [29]. Additionally, it was demonstrated that the Du/Egy/Benha/2020/DHAV-1 strain shares the same HVR amino acid sequence with several European, Chinese, and one Egyptian isolate. This suggests that evolution followed a similar path, indicating these strains may originate from a common ancestor. Previous studies have identified several conserved HVR profiles, with some patterns observed across different continents, underscoring the widespread dissemination and persistence of DHAV-1 lineages over long distances [30].

Therefore, genomic and phylogenetic analysis of the Du/Egy/Benha/2020/DHAV-1 strain is useful in understanding the current updates on the DHAV-1 and its adaptation to different geographical environments. The fact that DHAV-1 is closely related to strains from Hungary and China, and due to the low antigenic variability, it is evident that its genome remains rather stable. This stability may help the elaboration of efficient vaccines but the fact that specific mutations such as S178Y might contribute to immune evasion, call for constant surveillance of DHAV-1 evolution and cross neutralization studies.

Current vaccination efficacy is challenged by mutations in the DHAV-1 genome, particularly in the VP1 protein, which have a substantial impact on viral virulence and immune-escape mechanisms [31]. Since the VP1 protein includes the main neutralizing epitope and is necessary for viral attachment to host cells, its variability can alter antigenicity and impact antibody recognition across DHAV-1 strains [32]. The cross-neutralization results suggests that while the vaccine-induced antibodies effectively neutralized the vaccine strain, they elicited an even stronger response against the field isolate, potentially indicating antigenic similarities or enhanced immune recognition. On the contrary, antibodies generated against the field isolate demonstrated neutralizing titer of 2^7^ against both the vaccine and field strains, suggesting a broader but relatively balanced neutralization capacity. The comparable titers against both strains indicate that the field isolate-induced antibodies may confer cross-protection, albeit at a slightly lower level than the homologous response observed for the field strain. During evaluation of the virus cross-neutralization the R value of *R* ≤ 0.5 indicates a minor difference between the two strains that could be regarded to the genetic relation of the two viruses [23]. Moreover, our findings are consistent with the previous findings [31, 33] which confirmed the immune-protective efficacy of DHAV-1 vaccines against genotype-matched homologous field strains while showing a reduced capacity to neutralize divergent strains. Additionally, it was demonstrated that the VP1 gene sequence of the Egyptian vaccinal strain is genetically distinct from certain other Egyptian strains [34], which may explain the continuous emergence of DHAV-1 strains despite vaccination efforts.

## Conclusion

These findings effectively characterized the DHAV-1 strain isolated from Egypt, providing broad understandings to DHAV-1 genomic construction and antigenic properties. The close genetic relationship with strains from Hungary and China demonstrates a shared origin and highlights the worldwide dissemination of DHAV-1. The discovery of a new mutation, S178Y, in the VP1 protein underscores the capability of DHAV-1 for potential immune evasion, necessitating ongoing surveillance of viral evolution to maintain the effectiveness of current vaccinations. Further investigations and monitoring are needed to prevent the effects of DHAV-1 on poultry health around the globe and to guarantee the appropriateness of the existing and future vaccines. Additionally, further studies are needed to assess the potential cross-protection offered by these vaccines against other circulating DHAV genotypes, particularly DHAV-3, which lacks a commercially available vaccine in Egypt.

## Materials and methods

### Ethics approval and consent to participate

Clinical samples were collected from a privately owned duck farm with the informed consent of the owner. The study protocol was approved under the guidelines of the Institutional Animals Care and Use Committee (IACUC) Research Ethics Board, Faculty of Veterinary Medicine, Benha University with Approved Permission Number (BUFVTM 07-02-24).

### Clinical samples collection and preparation

The diseased ducklings were humanely sacrificed by cervical dislocation following standard ethical protocols. This method was chosen as it is a widely accepted humane technique for euthanizing small poultry species, ensuring a rapid and painless procedure. Liver and kidney samples (*n* = 20) were collected from young duck farm flocks, aged one week, showing signs of DVH infection in Benha, Egypt, during 2021. The affected flock was privately owned, and permission was obtained from the farm owner before sample collection. No experimental animals were used, as samples were collected from naturally infected ducklings during a disease outbreak. The affected birds had a significant mortality reached over 80% associated with ataxia and loss of balance triggered by opisthotonus. Necropsy showed severe petechial hemorrhages on the liver, along with swelling and congestion in the kidneys. The samples were homogenized in a 10% (w/v) phosphate-buffered saline solution containing antibiotics (penicillin 10,000 IU/ml and streptomycin 10 mg/ml). The homogenate was clarified by centrifugation at 5,000 rpm at 4 °C for 5 min, then filtered through a 45-µm filter and then stored at −80 °C until used for viral isolation and RT-PCR and viral isolation [35]. The other part was preserved in 10% neutral buffered formalin for histopathological examination.

The liver and kidney samples preserved in 10% neutral buffered formalin were embedded in paraffin. Sections, 5 μm thick, were stained using Harris’s haematoxylin and eosin. Histopathological changes were assessed based on criteria including congestion, haemorrhage, cytoplasmic vacuolation, necrosis, and inflammatory infiltration [36].

### RNA extraction, cDNA synthesis and RT-PCR

Total RNA was isolated from the supernatant of homogenate specimens using the QIAamp Viral RNA Mini Kit (Qiagen, GmbH, Hilden, Germany), following the manufacturer’s instructions. The RNA extracts were converted to cDNA using the QuantiTect Rev. Transcription Kit (Qiagen, GmbH, Hilden, Germany), as per the manufacturer’s instructions, and then stored at −20 °C until used. The RT-PCR assay was conducted to detect DHAV through amplifying of the VP1 gene as previously described by Doan et al., using the VP1 gene primer set: Forward: 5ʹ- GCCCCACTCTATGGAAATTTG-3ʹ, Reserve: 5ʹ-ATTTGGTCAGATTCAATTTCC-3ʹ [37]. The reaction protocol included three main steps: reverse transcription at 45 °C for 30 min, initial denaturation at 95 °C for 5 min, and 30 amplification cycles comprising denaturation at 95 °C for 30 s, annealing at 55 °C for 45 s, and extension at 72 °C for 45 s. A final extension was performed at 72 °C for 10 min. The PCR products were separated on a 1.2% agarose gel for visualization.

### Virus isolation in embryonated duck eggs

Viral Viral isolation was performed by inoculating 0.2 mL of liver homogenate supernatant into the allantoic sacs of 10–14-day-old embryonated duck eggs obtained from a non-vaccinated breeder flock (Meet al.-Attar, Benha, Egypt). The process was repeated three times through blind passages, following the guidelines of OIE, (2023). The eggs were incubated at 37 °C for 3–7 days, with daily candling to monitor embryonic mortality. The allantoic fluid was collected, and the isolated virus was verified by RT-PCR, titrated in the median egg infectious dose (EID_50_) according to Reed and Muench [38]. The allantoic fluid was kept at −80 °C until neutralization assay was performed.

### Complete genome sequencing and phylogenetic analysis

For next generation sequencing, 150 µl of allantoic fluid at the third passage was mixed with 750 µl of TRIzol (ABTizol) reagent for RNA extraction. RNA was isolated, quality-assessed, and converted to cDNA for library preparation, then sequenced at Eurofins Genomics, Germany. The resulting assembly was screened against available DHAV-1 sequences in GenBank. Contigs homologous to DHAV-1 were then used for further analysis.

The final genome sequences and its annotation were deposited in GenBank under the BioProject: PRJNA1028634, BioSample: SAMN37847919 and accession number OR738706.1for Du/Egy/Benha/2020/DHAV-1.

The VP0, VP1, and VP3 gene sequences were aligned using ClustalW and analysed in MEGA X [39] together with other DHAV-1 sequences obtained from the NCBI GenBank database. The phylogenetic trees were performed using maximum likelihood analysis with 1,000 bootstrap iterations. The Tamura-Nei model, was chosen as the most suitable model. Furthermore, the genetic relationship between Du/Egy/Benha/2020/DHAV-1 genes and corresponding viral sequences obtained from GenBank was evaluated. Consequently, the individual gene sequences were aligned using ClustalW and pairwise distances were computed for each gene. The distances were subsequently applied to generate a heatmap using the “heatmap” package in R (version 3.6.1). The antigenic determinants of the VP1 protein were explored using the approach of Kolaskar and Tongaonkar with the (Immune Epitope Database) IEDB Analysis Resource (iedb.org) [40]. Using the FASTA sequence of the structural VP1 protein in the IEDB Analysis Resource involves inputting the sequence into a selected prediction tool such as MHC Class I/II or B-cell epitope prediction followed by choosing the host species and relevant MHC alleles for T-cell analysis. The system then uses algorithms like NetMHC or consensus methods to identify peptide regions likely to bind MHC molecules or be recognized by B-cell receptors. Results include ranked epitopes with binding scores and can be further analyzed using tools for epitope conservancy, clustering, or population coverage.

### Virus neutralization assay

Hyperimmune sera were produced at the Veterinary Serum and Vaccine Research Institute (VSVRI), Agricultural Research Center (ARC), Cairo, Egypt, according to the reference protocols for antibody production against both the DHAV-1 field isolate and the live attenuated vaccine strain. The reference sera were kindly provided as a gift to support the completion of the study. The VN assay involved constant amounts of viruses and diluted serum. Briefly, 2-fold serial dilutions of inactivated hyperimmune sera were mixed with 10^2^ ELD_50_ of virus for 60 min at 37 ◦C. Subsequently, the serum–virus mixtures were inoculated into 9-day-old embryonated SPF chicken eggs (Kom Oshim, Al-Fayoum, Egypt) via the allantoic route. Controls included sera only and viruses only. The inoculated embryonic eggs were monitored daily, and dead embryos were noted up to 7–10 days post-inoculation. The neutralizing titers of antibodies were determined by the minimal serum dilution that prevented chicken embryo death, with mean values obtained from two repeated experiments. The antigenic relatedness (R value) between virulent and vaccine strains were calculated [41]. A result of 0.67 ≤ *R* ≤ 1.5 indicates no significant antigenic variation between the two viruses, whereas results of 0.5 ≤ *R* ≤ 0.67 indicates a minor difference. An R value < 0.5 indicates a significant difference between the tested viruses [42].

## Supplementary Information

Supplementary Material 1.

## Data Availability

The final genome sequences and its annotation were deposited in GenBank under the following: BioProject: PRJNA1028634: https://www.ncbi.nlm.nih.gov/bioproject/PRJNA1028634Accession number: OR738706.1: https://www.ncbi.nlm.nih.gov/nuccore/OR738706.1.
