# Supplementary figures and images for: Complete genome sequencing, molecular and antigenic characterization of duck hepatitis A virus type 1 isolated in Benha, Egypt
Source: BMC Vet Res. 2025 Oct 3;21:571. doi: 10.1186/s12917-025-05010-5 (PMC12492683; doi:10.1186/s12917-025-05010-5)

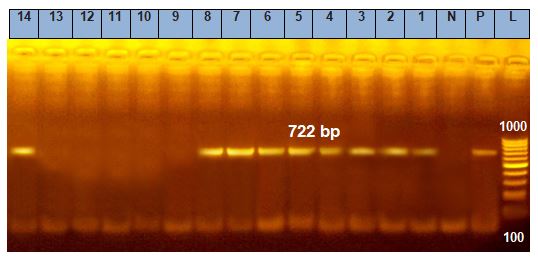

Supplement: Supplementary file 1 — Supplementary Material 1. [file 12917_2025_5010_MOESM1_ESM.jpeg]
